# High MLL2 expression predicts poor prognosis and promotes tumor progression by inducing EMT in esophageal squamous cell carcinoma

**DOI:** 10.1007/s00432-018-2625-5

**Published:** 2018-03-12

**Authors:** Abulajiang Abudureheman, Julaiti Ainiwaer, Zhichao Hou, Madiniyat Niyaz, Abdugheni Turghun, Ayshamgul Hasim, Haiping Zhang, Xiaomei Lu, Ilyar Sheyhidin

**Affiliations:** 1 Department of Thoracic Surgery, First Affiliated Hospital of Xinjiang Medical University, Urumqi, 830054 Xinjiang Uygur Autonomous Region People’s Republic of China; 2 Clinical Medical Research Institute, First Affiliated Hospital of Xinjiang Medical University, Urumqi, 830054 Xinjiang Uygur Autonomous Region People’s Republic of China; 3 Department of Pathology of Xinjiang Medical University, Urumqi, 830054 Xinjiang Uygur Autonomous Region People’s Republic of China

**Keywords:** MLL2/KMT2D, Esophageal squamous cell carcinoma (ESCC), Prognosis, EMT, TGF-β/Smad

## Abstract

**Background:**

MLL2 has been identified as one of the most frequently mutated genes in a variety of cancers including esophageal squamous cell carcinoma (ESCC). However, its clinical significance and prognostic value in ESCC has not been elucidated. In the present study, we aimed to investigate the expression and role of MLL2 in ESCC.

**Methods:**

Immunohistochemistry (IHC) and qRT-PCR were used to examine the expression profile of MLL2. Kaplan–Meier survival analysis and univariate and multivariate Cox analyses were used to investigate the clinical and prognostic significance of MLL2 expression in Kazakh ESCC patients. Furthermore, to evaluate the biological function of MLL2 in ESCC, we applied the latest gene editing technique CRISPR/Cas9 to knockout MLL2 in ESCC cell line Eca109. MTT, colony formation, flow cytometry, scratch wound-healing and transwell migration assays were performed to investigate the effect of MLL2 on ESCC cell proliferation and migration. The correlation between MLL2 and epithelial–mesenchymal transition (EMT) was investigated by Western blot assay in vitro and IHC in ESCC tissue, respectively.

**Results:**

Both mRNA and protein expression levels of MLL2 were significantly overexpressed in ESCC patients. High expression of MLL2 was significantly correlated with TNM stage (*P* = 0.037), tumor differentiation (*P* = 0.032) and tumor size (*P* = 0.035). Kaplan–Meier survival analysis showed that patients with low MLL2 expression had a better overall survival than those with high MLL2 expression. Multivariate Cox analysis revealed that lymph node metastasis and tumor differentiation were independent prognostic factors. Knockout of MLL2 in Eca109 inhibited cell proliferation and migration ability, induced cell cycle arrest at G1 stage, but it had no significant effect on apoptosis. In addition, knockout of MLL2 could inhibit EMT by up-regulation of E-Cadherin and Smad7 as well as down-regulation of Vimentin and p-Smad2/3 in ESCC cells. In cancer tissues, the expression of E-Cadherin was negatively correlated with MLL2 expression while Vimentin expression was positively correlated with MLL2 expression.

**Conclusion:**

Our results indicate that overexpression of MLL2 predicts poor clinical outcomes and facilitates ESCC tumor progression, and it may exert oncogenic role via activation of EMT. MLL2 may be used as a novel prognostic factor and therapeutic target for ESCC patients.

## Introduction

Esophageal cancer is one of the most common malignant tumors with high incidence and mortality worldwide (Ferlay et al. 2015). Esophageal squamous cell carcinoma (ESCC) accounts for the most of the esophageal cancers and is the fourth leading cause of death from cancer in China (Lin et al. 2013). Xinjiang is one of the high-risk areas in China, where the incidence of ESCC in Kazakh minority is significantly higher than the national average (Zheng et al. 2010). Despite the advances in treatment of ESCC, the 5-year overall survival rate is still very poor. Deep invasion and metastasis are main reasons for poor prognosis of ESCC and it is important to elucidate the underlying mechanisms to improve the outcomes of ESCC patients.

MLL2 (also known as KMT2D/ALR/MLL4), which is located to 12q12-13, encodes a histone methyltransferase that is mainly responsible for the methylation of histone H3 lysine 4 (H3K4), and plays an important role in epigenetic regulation of gene transcription (Bögershausen et al. 2013; Ruthenburg et al. 2007). Recently, many exome sequencing studies have revealed the MLL2 gene as one of the most frequently mutated genes in a variety of human cancers, including follicular lymphoma, diffuse large B-cell lymphoma, renal carcinoma, prostate cancer, bladder carcinoma, gastric carcinoma, breast cancer, lung carcinomas (Dalgliesh et al. 2010; Grasso et al. 2012; Gui et al. 2011; Morin et al. 2011; Pasqualucci et al. 2011; Pleasance et al. 2009; Stephens et al. 2012; Zang et al. 2012), suggesting that MLL2 may play an important role in tumorigenesis in a variety of tumors. As most of the mutations were inactivated and predicted to produce protein products without the key methyltransferase domain, it was considered as a tumor-suppressor (Morin et al. 2011; Parsons et al. 2011; Pasqualucci et al. 2011). However, studies on its role in some cancers showed contradictive results that whether it is an oncogene or tumor-suppressor gene remains to be further elucidated (Guo et al. 2013; Issaeva et al. 2007; Zhang et al. 2015).

MLL2 was also found to be frequently mutated in ESCC and conjectured as a tumor suppressor due to the inactivated mutations (Gao et al. 2014; Song et al. 2014). However, the role of MLL2 in ESCC remains unknown. In this study, we examined the expression level of MLL2 and evaluated its prognostic value in ESCC patients. Moreover, we knocked out MLL2 in Eca109 cells by CRISPR/Cas9 gene editing system to further explore the role of MLL2 and the possible mechanism underlying its involvement in ESCC cell progression, and further confirmed the result of in vitro study by IHC in cancer tissues.

## Materials and methods

### Patients and samples

To investigate the mRNA levels and protein expression of MLL2 in Kazakh patients with ESCC, we selected 42 samples for qRT-PCR and 67 samples for immunohistochemistry (IHC), respectively. All the patients underwent curative surgical resection at the department of Thoracic Surgery of the First Affiliated Hospital, Medical University of Xinjiang, China, and confirmed by histopathology. For PCR, the tissue samples were obtained during surgery and frozen immediately after resection, then stored at − 80 °C until use. Paraffin-embedded ESCC tissue sections were acquired for IHC from the pathology Department. Both tumor samples and matched adjacent normal tissues (≥ 5 cm away from the tumor) were available for each patient. None of the patients received preoperative chemotherapy, radiotherapy or other cancer-related treatments. The disease stage of the ESCC patients were determined based on the TNM classification of AJCC Cancer Staging Manual (7th edition). Other relevant clinicopathological information was available for all the patients. All patients were enrolled with written informed consents, and the study was approved by the Ethical Committee of the Affiliated Hospital of Xinjiang Medical University.

Due to the limitation of samples, for the IHC staining of E-cadherin, Vimentin and Smad7, we selected 26 samples for each group, respectively. All the 26 cases were among the ESCC samples mentioned above that used for MLL2 expression.

### RNA Extraction and qRT-PCR

The total RNA was extracted from fresh frozen tissues with TRIzol reagent (Invitrogen Life Technologies, CA, USA). Purity and concentration of RNA were detected by NanoDrop ND 1000. RNA was reversely transcribed into cDNA using prime SCRIPT™ RT-PCR kit (TaKaRa, Dalian, China). The qRT-PCR analysis was performed on an IQ5 system (Bio-Rad, USA) with SYBR Green reagents (Boster, Wuhan, China) according to the manufacturer’s instructions. β-actin was used as an internal reference for normalization and the relative expression of MLL2 mRNA was evaluated by the 2^− ΔΔCT^ method. The primers used in this study were as follows: MLL2, forward: 5′-TGACAAGTGTGAATCCCGTGAAG-3′ and reverse: 5′-AACCATTTCATCCGTTGTTACG AAG-3′; β-actin, forward: 5′-ATGATGATATCGCCGCGCTC-3′ and reverse: 5′-TCGATGGG GTACTTCAGGG-3′.

### Immunohistochemistry (IHC)

Protein expression of MLL2, E-Cadherin, Vimentin and Smad7 was performed by the IHC and it was followed as previously described (Gambichler et al. 2016). In brief, 4 µm tissue sections were cut from the paraffin-embedded blocks and transferred to glass slides. Then the slides were incubated in 60 °C for 1 h, followed by deparaffinization and hydration with xylol and gradient alcohol, respectively. Then the antigen retrieval step was performed by heating in a microwave oven at high fire mode for 5 min in citrate buffer (pH 6.0). After cooled at room temperature, the sections were treated with 3% hydrogen peroxide for 10 min to remove the endogenous peroxidase. Subsequently, anti-MLL2 goat polyclonal antibody (ab15962, Abcam, Cambridge, USA; dilution 1:100), anti-E-Cadherin mouse monoclonal antibody (ab76055, Abcam; dilution 1:300), anti-Vimentin rabbit monoclonal antibody (ab76055, Abcam; dilution 1:500), anti-Smad7 mouse monoclonal antibody (ab55493, Abcam; dilution 1:500) were used to incubate the sections as the primary antibody overnight at 4 °C in a moist chamber. Polink-2 plus HRP Detection Kit was used as the secondary antibody following the manufacturer’s instructions. The sections were washed with TBST (Tris-buffered saline with Tween) for 3 × 5 min after each step mentioned above. Finally, DAB was used to visualize immunoreactivity and counterstained with hematoxylin. The primary antibody was replaced by TBST as negative control.

IHC staining results were blindly evaluated by two independent pathologists. For MLL2, nuclear staining of MLL2 was considered as positive. And > 50% positive tumor nuclei was defined as high expression while ≤ 50% positive tumor nuclei was defined as low expression.

For E-cadherin, Vimentin and Smad7, the IHC staining was scored by combination of the percentage and intensity of positively stained tumor cells (Goumans et al. 2014). The scoring criterion for percentage of positive stained cells was as follows: 0, < 5%; 1, 6–25%; 2, 26–50%; and 3, > 50%. The scoring criterion for staining intensity: 0, negative; 1, weak staining; 2, moderate staining; and 3, strong staining. The final score was determined by multiplying the percentage score and the staining intensity score (the total score ranging from 0 to 9).

### Cell culture and transfection

The human ESCC cell line Eca109 was purchased from WuHan University (Hubei, WuHan, China) and cultured in RPMI-1640 medium plus 10% fetal bovine serum and penicillin and streptomycin in a 5% CO_2_ humidified incubator at 37 °C.

CRISPR/Cas9 genome-editing technique can induce frame shift mutations at specific sites in the genome through a synthetic sgRNA that result in a loss-of-function allele. And it has been reported to be a very efficient way to knock out genes (Ran et al. 2013; Shalem et al. 2014). To investigate the biological role of MLL2 in ESCC, we used the CRISPR/Cas9 gene editing system to generate MLL2-knockout cells in ESCC cell line Eca109. First, Eca109 cells were transfected with Lenti-Cas9 lentivirus and screened with puromycin to select the cells that stably expressed Cas9 after 3 days of transfection. Then, the selected cells were transfected with the sgRNA lentivirus and the EGFP expression was detected with a fluorescent microscope (Olympus, Tokyo, Japan). The cells were collected for further experiment when the transfection efficiency reached greater than 80%. The detailed CRISPR/Cas9 knockout procedure is described in supplementary material.

### MTT assay

MTT assay was used to evaluate the effect of MLL2 knockout on cell proliferation of Eca109 cell line. Briefly, cells were seeded into a 96-well plate at a concentration of 2000 cells/well. After growing for 24, 48, 72, 96 and 120 h, cells were treated with 20 µl MTT solution (Genview, JT343, 5 mg/ml). The medium was removed and replaced with 100 µl DMSO to dissolve formazan precipitates after incubated at 37 °C for 4 h. Then the OD value at 490 nm was measured with a microplate reader (Tecan infinite, M2009PR).

### Colony formation assay

Cells were inoculated in 6-well plates at a density of 600 cells/well and the culture medium was replaced every 3 days. After growing for 10 days, cells were fixed with 4% paraformaldehyde and stained with Giemsa staining solution (Dingguo Biotechnology Co., Ltd. Shanghai). The number of clones was counted manually.

### Apoptosis assay and cell cycle analysis

The apoptosis assay was detected by Annexin V-APC (eBioscience, cat.No. 88-8007) single staining method in the fifth day after transfection. Briefly, cells were washed with cold D-Hanks solution and binding buffer, respectively. Then cells were resuspended with 200 µl binding buffer and incubated for 15 min at room temperature avoided light after adding 10 µl Annexin V-APC. Cell apoptosis of the stained cells were measured by flow cytometry (Millipore, Guava easyCyte HT).

For cell cycle analysis, cells were washed with cold D-Hanks solution and fixed with 75% cold ethanol for 2 h. Then the cells were washed and resuspended with staining solution containing propidium iodide (PI, 50 µg/ml) and RNase (50 µg/ml). The cell cycle was analyzed by flow cytometry and the distribution of different phases (G1, S and G2/M) was measured.

### Scratch wound-healing assay and migration assay

Wound-healing assay and transwell migration assay were used to assess the cell migration ability. For wound-healing assay, cells (5 × 10^4^) were seeded in a 96-well plate and grew till they reached more than 90% confluence. A 96-well mechanical floating pin tool (VP Scientific, VP-408FH) was used to make the scratches. A fluorescent microscope (Olympus, Tokyo, Japan) was used to take the images at appropriate time (0, 24, 48 h).

For transwell migration assay, cells (1 × 10^5^) were seeded in the upper chamber of a 24-well plate (Corning) and cultured with 100 µl serum-free medium. 600 µl of culture medium containing 30% FBS was added to the lower chamber. After incubated for 36 h, the non-migrated cells that remained in the upper chamber were erased by a cotton swab. The migrated cells in the lower chamber were fixed and stained with Giemsa. Pictures of nine random fields (magnification, × 200) were taken with an inverted microscope and counted. ALL the experiments were repeated three times.

### Western blotting assay

After cells were harvested, the total proteins were extracted with 2 × Lysis Buffer and the protein concentrations were determined by a BCA Protein Assay Kit (Beyotime Biotechnology, China). Equal amounts of proteins (30 µg) were separated with 10% SDS–PAGE and then electrophoretically transferred to PVDF membrane (Millipore, USA). The membrane were blocked with TBST solution containing 5% skimmed milk for 1 h at room temperature and incubated with primary antibodies (E-Cadherin: Abcam, ab76055, dilution 1:500; Vimentin: Abcam, ab92547, dilution 1:500; Smad7: Abcam, ab55493, dilution 1:500; Smad2/3: Abcam, ab202445, dilution 1:500; p-Smad2/3: Abcam, ab63399, dilution 1:500; GAPDH: Santa Cruz, sc-32233, dilution 1:2000) at 4 °C overnight. They were then incubated with proper secondary antibodies for 1.5 h at room temperature. The proteins were detected with an ECL western blotting substrate kit (Pierce).

### Statistical analysis

SPSS 21.0 software (SPSS Inc., Chicago, IL) and GraphPad Prism 5.0 were used for data analysis and figure process. The results of the experiments were presented as mean ± SEM. Chi-square test or Fisher’s exact test, one-way analysis of variance, as well as Student’s t-test were used as appropriate. The survival time started from the date of surgery to death or the last follow-up date. Survival analysis was evaluated by Kaplan–Meier and log-rank test. Further multivariate analysis was performed by Cox proportional hazards regression model to identify the independent risk factors for ESCC. All the analyses were two-sided test and considered statistically significant at a *P* < 0.05 level.

## Results

### Expression of MLL2 is up-regulated and associated with clinicopathological factors in ESCC patients

**Fig. 1:**
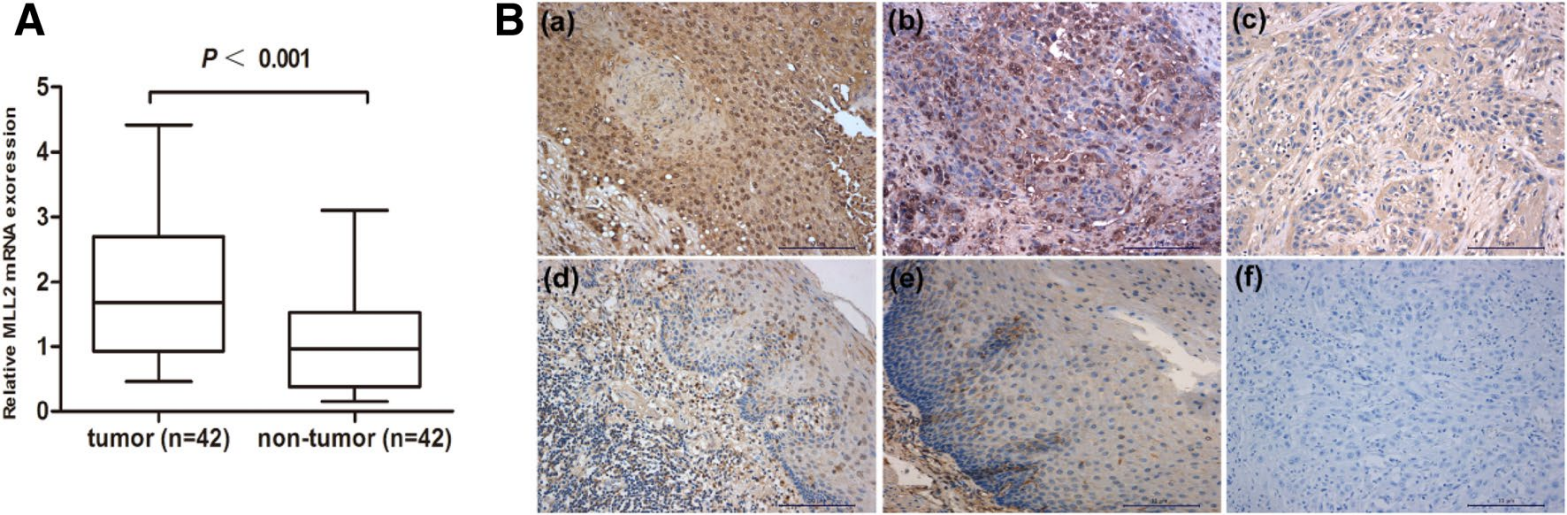
MLL2 expression in ESCC. **a** MLL2 mRNA expression was examined in 42 ESCC (tumor) and paired adjacent normal tissues (non-tumor) by qRT-PCR and MLL2 expression was up-regulated in ESCC (*P* < 0.001). **b** MLL2 expression in ESCC and adjacent normal tissues by IHC (× 200). All samples stained for MLL2 additionally displayed low-to-moderate levels of cytoplasmic immunoreactivity. Positive staining of MLL2 in ESCC tissues: **a** high expression of MLL2 in ESCC (> 50% positive nuclear staining); **b** low expression of MLL2 in ESCC (≤ 50% positive nuclear staining). **c** Negative MLL2 staining in ESCC tissues. **d** Low expression of MLL2 in adjacent normal tissues. **e** Negative staining of MLL2 in adjacent normal tissues. **f** Negative control

First, the mRNA expression of MLL2 was assessed by qRT-PCR in 42 ESCC tissues and paired adjacent normal tissues. As shown in Fig. 1a, mRNA expression of MLL2 was significantly up-regulated in ESCC compared with adjacent normal tissues (*P* < 0.001). Then, we examined the protein expression status of MLL2 by IHC. The staining results indicate that MLL2 mainly expressed in nuclear, cytoplasmic staining was considered non-specific and not included in the evaluation (Juhlin et al. 2015) (Fig. 1b). Among the 67 ESCC tissues, high expression rates of MLL2 in tumor and adjacent normal tissues were 43.3% (29/67) and 11.9% (8/67), respectively (Table 1). A significant overexpression of MLL2 was found in tumor tissues in contrast to adjacent normal tissues (*P* < 0.05).

**Table 1:** The correlation between MLL2 expression and clinicopathological characteristics of Kazakh ESCC patients

Characteristics	*n*	MLL2 expression		χ^2^	*P*
		High (%)	Low (%)		
Specimen				16.465	< 0.001*
Adjacent normal tissue	67	8 (11.9)	59 (88.1)		
ESCC	67	29 (43.3)	38 (56.7)		
Gender				0.448	0.348
Female	19	7 (36.8)	12 (63.2)		
Male	48	22 (45.8)	26 (54.2)		
Age (years)				2.417	0.095
> 60	35	12 (34.3)	23 (65.7)		
≤ 60	32	17 (53.1)	15 (46.9)		
TNM stage				4.424	0.037*
I–II	54	20 (37.0)	34 (63.0)		
III	13	9 (69.2)	4 (30.8)		
T classification				1.123	0.209
T1–T2	28	10 (35.7)	18 (64.3)		
T3–T4	39	19 (48.7)	20 (51.3)		
*N* classification				0.781	0.264
No	41	16 (39.0)	25 (61.0)		
Yes	26	13 (50.0)	13 (50.0)		
Differentiation				4.447	0.032*
Well	18	4 (22.2)	14 (77.8)		
Moderately/poorly	49	25 (51.0)	24 (49.0)		
Tumor size				4.195	0.035*
< 4	35	11 (31.4)	24 (68.6)		
≥ 4	32	18 (56.3)	14 (43.8)		
Vascular invasion				2.2	0.118
Negative	52	20 (38.5)	32 (61.5)		
Positive	15	9 (60.0)	6 (40.0)		

*Statistically significant (*P* < 0.05); *n * number of cases;* MLL2* Expression cases (Ratio); *Χ*^2^ Chi-square value

We further examined the correlation of MLL2 protein expression and the clinicopathological characteristics as shown in Table 1. The results demonstrated that high MLL2 expression was significantly correlated with TNM stage, tumor differentiation and tumor size (*P* < 0.05). No positive correlations were found between other clinicopathological parameters and MLL2 expression.

### High expression of MLL2 predicts poor prognosis in ESCC patients

**Fig. 2:**
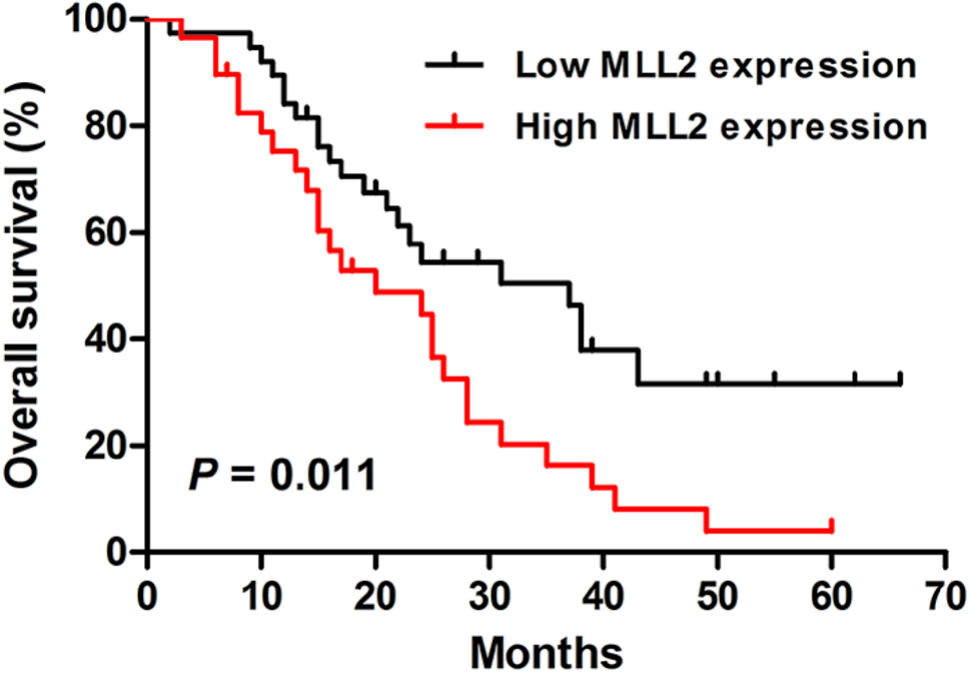
Kaplan–Meier analysis of overall survival for Kazakh ESCC patients. Patients with low MLL2 expression had a better overall survival than those with high MLL2 expression (*P* = 0.011, Log-rank test)

The statistical analysis of overall survival was performed by Kaplan–Meier method. The result showed that the patients with low MLL2 expression had a better prognosis than those with high MLL2 expression (*P* = 0.011, Log-rank test, Fig. 2). In addition, the univariate Cox regression analysis showed that lymph node metastasis, depth of invasion, tumor differentiation and MLL2 expression were significantly associated with overall survival of ESCC patients (Table 2). Multivariate Cox analysis was used to further evaluate the prognostic factors of ESCC and the results revealed that lymph node metastasis and tumor differentiation were independent prognostic factors.

**Table 2:** Univariate and multivariate analysis of the overall survival in ESCC

Variables	Univariate analysis		Multivariate analysis	
	HR (95% CI)	*P* value	HR (95% CI)	*P* value
Gender				
Female vs. male	0.713 (0.369–1.377)	0.314		
Age				
< 60 vs. ≥ 60	0.604 (0.337–1.081)	0.090		
Lymph node metastasis				
Negative vs. positive	0.377 (0.206–0.687)	0.001^*^	0.425 (0.225–0.802)	0.008*
Depth of invasion				
T1–T2 vs. T3–T4	0.531 (0.287–0.981)	0.043^*^	0.513 (0.274–0.960)	0.051
Differentiation				
Well vs. moderately/poorly	3.425 (1.546–7.586)	0.002^*^	0.403 (0.173–0.940)	0.035^*^
Tumor size				
< 4 cm vs. ≥ 4 cm	1.564 (0.869–2.815)	0.136		
Vascular invasion				
Negative vs. positive	2.011 (0.967–4.050)	0.058		
MLL2 expression				
Low vs. high	2.085 (1.163–3.736)	0.014*	0.601 (0.324–1.115)	0.089

*Statistically significant (*P* < 0.05)

*HR* hazard ratio, *CI* confidence interval

### Knockout of MLL2 suppresses ESCC cell proliferation in vitro

**Fig. 3:**
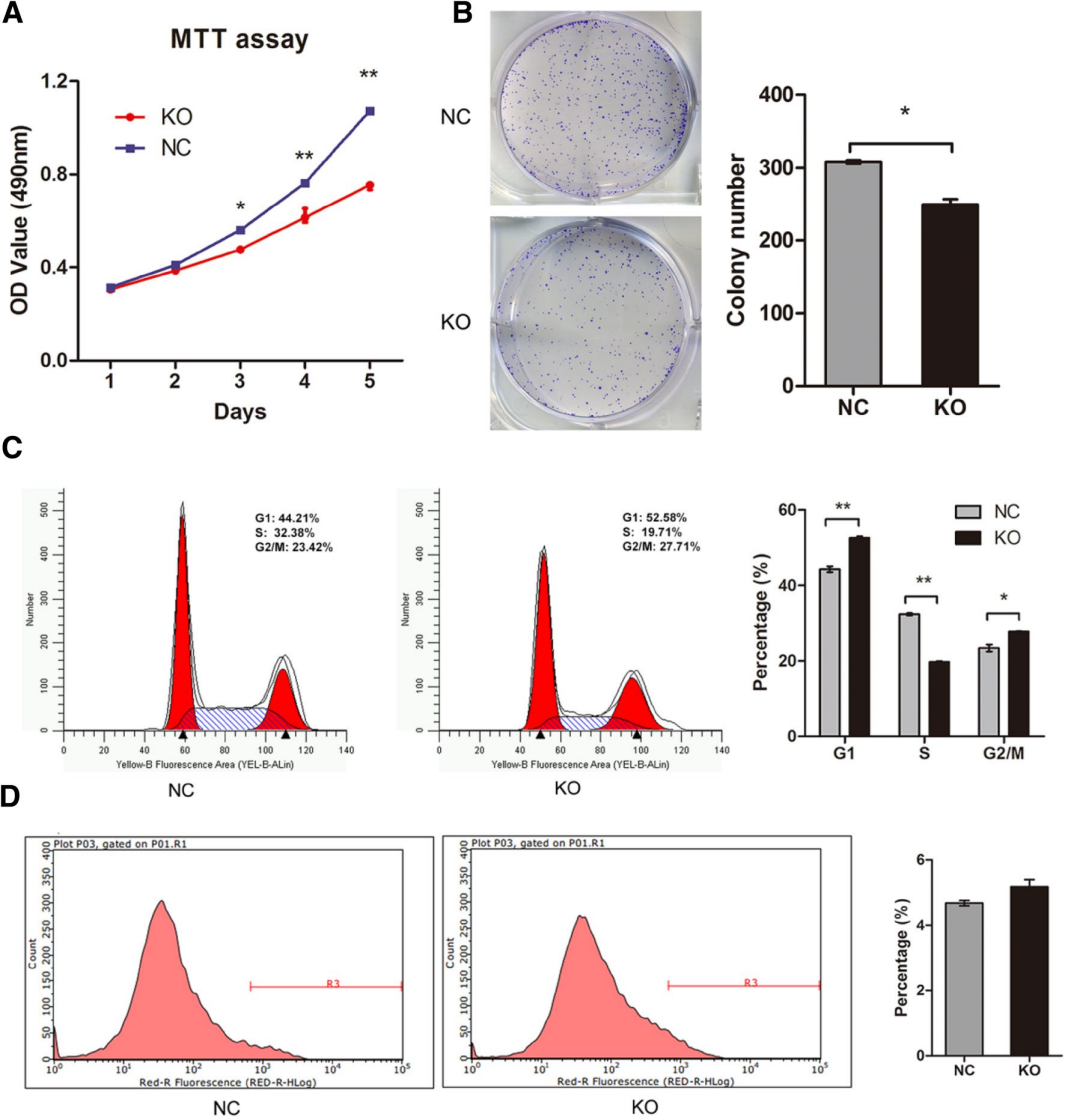
Effect of MLL2 knockout on cell proliferation in Eca109. **a, b** MTT assay and colony formation assay showed that knockout of MLL2 significantly reduced the proliferation ability of Eca109 cells. **c** Effect of MLL2 knockout on cell cycle in Eca109. Knockout of MLL2 arrested cell cycle at G1 phase. **d** Effect of MLL2 knockout on cell apoptosis. Knockout of MLL2 did not significantly affected cell apoptosis in Eca109 (*P* > 0.05). NC, negative control. KO, MLL2 knockout group. *P < 0.05, **P < 0.01

The effect of MLL2 on Eca109 cell proliferation was determined. The MTT assay and colony formation assay results showed that knockout of MLL2 significantly reduced the proliferation ability of Eca109 cells compared with negative control (*P* < 0.05, Fig. 3a, b). Furthermore, we assessed the effect of MLL2 on cell apoptosis and cell cycle by flow cytometry. As shown in Fig. 3c, MLL2 knockout significantly changed cell cycle distribution. The percentage of the cells in S phase was significantly decreased (KO, 19.71%; NC, 32.38%; *P* < 0.01) while it was increased in G1 phase (KO, 52.58%; NC, 44.21%, *P* < 0.01) in the MLL2 knockout cells compared with the negative control group. In addition, knockout of MLL2 slightly increased the apoptosis rate of Eca109 cells, but there was no statistical difference between the two groups (*P* > 0.05, Fig. 3d). This result indicated that knockout of MLL2 could inhibit cell cycle progression by inducing cell cycle arrest at G1 stage, but did not significantly alter cell apoptosis. Taken together, these data suggested that knockout of MLL2 suppressed proliferation of ESCC cells.

### Knockout of MLL2 inhibits ESCC cell migration

**Fig. 4:**
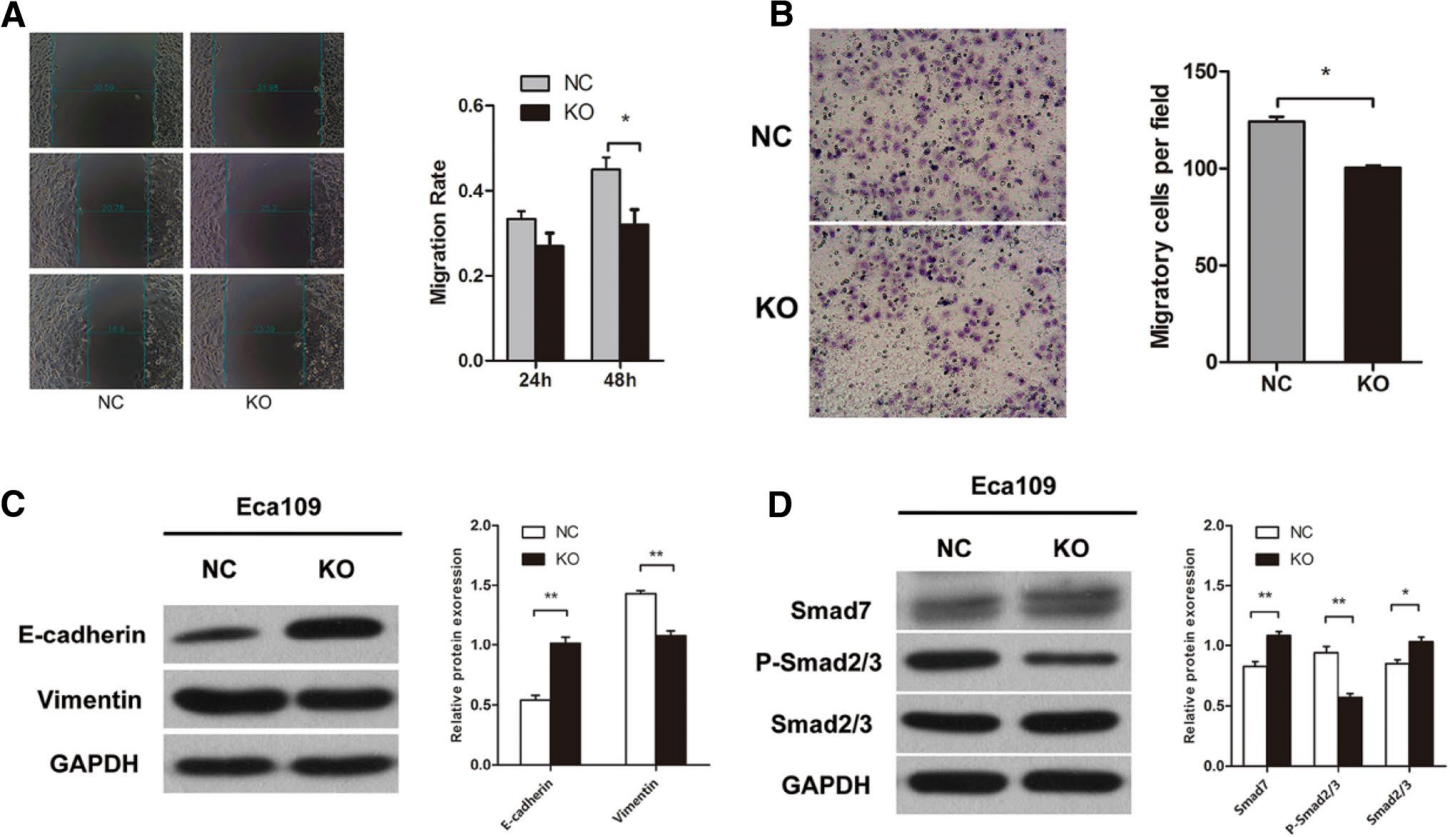
Effect of MLL2 knockout on cell migration and EMT in Eca109. **a** Scratch wound-healing assay and **b** tranwell migration assay showed that knockout of MLL2 significantly attenuated the migration ability of Eca109 cells. **c, d** Western blot analysis showed that knockout of MLL2 suppressed EMT in Eca109 cells. NC, negative control. KO, MLL2 knockout group. *P < 0.05, **P < 0.01

We used scratch wound-healing assay and transwell migration assay to examine the effect of MLL2 on cell migration. First, we performed wound-healing assay and the results showed that knockout of MLL2 significantly attenuated the migration ability of Eca109 cells (*P* < 0.05, Fig. 4a). The transwell migration assay also revealed that MLL2 knockout group markedly weakened the migration ability of Eca109 cells (*P* < 0.01, Fig. 4b), further confirmed the result of wound-healing assay. These results indicated that MLL2 could promote ESCC cell migration in vitro.

### MLL2 facilitates EMT in ESCC in vitro

To determine whether MLL2 was associated with EMT in ESCC cells, we examined the expression of EMT-related genes (E-Cadherin and Vimentin) by Western blot. The results showed that knockout of MLL2 significantly increased the expression of E-Cadherin and decreased the expression of Vimentin (*P* < 0.01, Fig. 4c). These results suggested that MLL2 may induce EMT in ESCC cells. TGF-β/Smad signaling pathway is a key inducer of EMT. We further examined the expression of the genes involved in the TGF-β/Smad signaling pathway (Smad7, Smad2/3 and p-Smad2/3) to determine whether MLL2 mediated EMT via Smad signaling pathway. The results showed that the expression of Smad7 and Smad2/3 was markedly increased, while p-Smad2/3 expression was decreased in MLL2 knockout group than the control group (*P* < 0.01, Fig. 4d). Taken together, these data suggested that MLL2 may induce EMT in ESCC cells via activating the TGF-β/Smad signaling pathway.

### MLL2 expression were associated with EMT in ESCC tissue

To confirm the results of the in vitro study, we further investigated the correlation of MLL2 expression and EMT in ESCC tissues. Among the 26 cases used for IHC staining of E-Cadherin, Vimentin and Smad7, there were 11 patients with MLL2 high expression and 15 patients with MLL2 low expression. We calculated the mean score of IHC staining of the above proteins in each group and evaluated their correlation with MLL2 expression. High IHC staining score represented high expression.

**Fig. 5:**
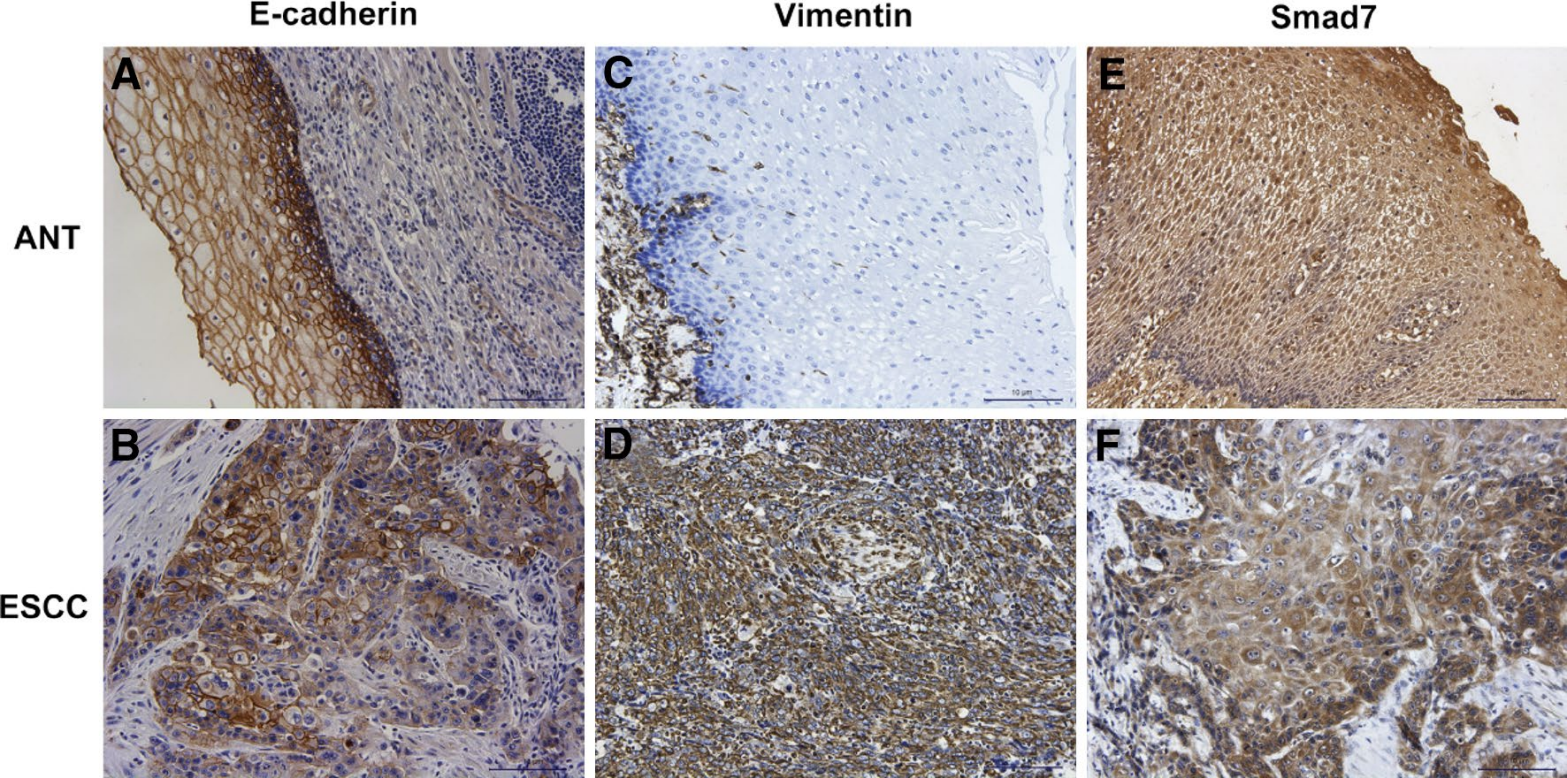
Representative immunohistochemical staining for E-cadherin (**a, b**); Vimentin (**c, d**); and Smad7 (**e, f**) in ESCC and adjacent normal tissues (ANT) (× 200)

Representative IHC staining images of E-Cadherin, Vimentin and Smad7 were shown in Fig. 5. The positive staining of E-Cadherin was mainly located in the membrane (Fig. 5a, b). And E-cadherin expression was significantly down-regulated in ESCC. Moreover, the expression of E-cadherin in MLL2 low expression group was significantly higher than MLL2 high expression group (Table 3). The positive staining of Vimentin was mainly located in the cytoplasm (Fig. 5c, d). In contrast to E-cadherin expression, Vimentin was significantly up-regulated in ESCC. And the expression of Vimentin in MLL2 high expression group was significantly higher than MLL2 low expression group (Table 3). These results indicated that MLL2 was positively associated with EMT in ESCC, consistent with the results of in vitro study by Western blot.

**Table 3:** The expression of E-Cadherin, Vimentin, Smad7 and their correlation with MLL2 expression in ESCC

	ESCC	ANT	*P*	MLL2 expression (Cases)		*P*
				High (11)	Low (15)	
E-cadherin	2.84 ± 1.37	5.60 ± 2.12	*P* < 0.001^*^	1.91 ± 0.83	3.57 ± 1.28	*P* < 0.001*
Vimentin	5.32 ± 2.13	1.76 ± 1.30	*P* < 0.001^*^	6.82 ± 2.23	4.14 ± 1.09	*P* < 0.005*
Smad7	3.36 ± 1.58	4.52 ± 1.50	*P* = 0.011^*^	2.72 ± 1.35	3.86 ± 1.61	*P* = 0.069

*Statistically significant (*P* < 0.05)

*ANT* adjacent normal tissues

As for Smad7, the positive staining was observed both in the cytoplasm and nucleus (Fig. 5e, f). The expression of Smad7 was significantly down-regulated in ESCC. However, its expression did not show significantly difference between MLL2 high expression and low expression group (Table 3).

## Discussion

In the present study, we examined the expression status of MLL2 in ESCC patients and found that both mRNA and protein levels of MLL2 exhibited significantly higher expression in tumor tissues than adjacent normal tissues. The high expression of MLL2 was closely associated with worse clinical outcomes in ESCC patients. In addition, MLL2 promoted the proliferation and migration abilities of ESCC cells by inducing EMT.

The extensive mutation of MLL2 suggests that it may be involved in the development of various cancers. Zhang et al. (2015) found that knockdown of MLL2 at the early stage of B cell development could lead to an increase in germinal-center (GC) B cells and enhanced B cell proliferation in mice, ultimately resulted in the occurrence of GC-derived lymphomas similar to human tumors, suggesting a tumor suppressor role for MLL2. However, studies in solid tumors such as breast and colorectal cancer emerged contradictory results. Knockdown of MLL2 in Hela cells significantly altered the growth characteristics resulting in reduced proliferation and migration capacity, and decreased tumorigenicity in mice (Issaeva et al. 2007). Another study involving colorectal and medulloblastoma cancer cell lines showed a similar result (Guo et al. 2013). These studies collectively indicate that MLL2 may have distinct roles in different tumors and its biological consequences are dependent on cancer type.

MLL2 has been found to be involved in tumor progression and associated with poor prognosis in several cancers. However, to our knowledge, the clinical significance and biological function of MLL2 in ESCC remains unknown. Juhlin et al. (2015) found that MLL2 expression was up-regulated in pheochromocytoma (PCC) compared to normal adrenals, and overexpression of MLL2 positively affected cell migration. In addition, PCCs with MLL2 mutations exhibited significantly larger tumor size than those with other gene mutations. Another study in gastrointestinal diffuse large B-cell lymphoma showed that high expression of MLL2 was associated with higher clinical stage and poor patient survival (Ye et al. 2015). High level of MLL2 was also associated with poor prognosis in breast cancer (Kim et al. 2014). In consistent with these results, we also found that MLL2 expression was significantly higher in tumor tissues than adjacent normal tissues in ESCC patients. And the high expression of MLL2 was correlated with TNM stage, tumor differentiation and tumor size. On the other hand, though other malignancy risk factors such as tumor invasion, lymph node metastasis and vascular invasion showed no significant relation with MLL2 expression, there was a tendency that patients with deeper invasion, lymph node metastasis and vascular invasion appeared to have a higher expression rate of MLL2. These results indicated that MLL2 may be involved in tumor malignancy in ESCC. In addition, we found that MLL2 expression was negatively associated with patient survival. Patients with high MLL2 expression had significantly poorer overall survival than those with low MLL2 expression, suggesting that high MLL2 expression may serve as a predictive marker of poor prognosis and may be a potential therapeutic target in ESCC.

To further explore the biological role of MLL2 in Eca109 cells, we used the CRISPR/Cas9 gene editing system to knock out MLL2 in Eca109 cells. Then we investigated the effects of MLL2 on Eca109 cell proliferation and migration. The results showed that knockout of MLL2 significantly reduced the proliferation ability of Eca109 by arresting the cell cycle in the G1 phase rather than affecting cell apoptosis. The results of scratch wound-healing assay and tranwell migration assay also indicated that knockout of MLL2 attenuated the migration ability of Eca109 cells, consistent with the previous studies (Guo et al. 2013; Issaeva et al. 2007). Therefore, knockout of MLL2 inhibited the cell growth and migration of Eca109 cells. In other words, overexpression of MLL2 may promote cell growth and metastasis in ESCC cells, which may lead to the unfavorable prognoses in ECSS patients.

EMT has been confirmed to play an important role in tumor progression and TGF-β/Smad signaling is a key inducer of EMT (Xu et al. 2009). Guo et al. (2013) found that knockout of MLL2 in colorectal cancer cells could lead to altered expression of a variety of genes, including the decreased expression of Vimentin and increased expression of Smad7, which is the negative regulator of TGF-β/Smad signaling through blocking the phosphorylation of Smad2/3 (p-Smad2/3) (Luo et al. 2014). Therefore, we speculated that MLL2 might promote ESCC cell metastasis through EMT via regulating the Smad signaling pathway. Our data showed that the expression levels of E-Cadherin and Smad7 were increased while the expression levels of Vimentin and p-Smad2/3 were decreased in MLL2 knockout group than the control group, which illustrated that knockout of MLL2 attenuated the EMT process and inhibited the TGF-β/Smad signaling. The IHC results in tissue also showed that MLL2 expression was inversely correlated with E-cadherin and positively correlated with Vimentin. Though Smad7 expression did not differ significantly between MLL2 high expression and low expression group, the expression score of Smad7 was higher in MLL2 low expression group than MLL2 high expression group, consistent with the results of in vitro study. These results indicated that MLL2 might induce EMT through activating the TGF-β/Smad signaling pathway, and contribute to the subsequent cancer progression in ESCC. The possible underlying mechanism might be that MLL2 down-regulated Smad7 expression, which led to the hyperactivation of TGF-β/Smad signaling and the promotion of cancer progression.

However, our sample size was relatively small, and the patients enrolled our study only included Kazakh minority patients. Therefore, further studies involving more samples and different ethnic groups as well as more ESCC cell lines would be necessary to further validate our results.

In summary, we found that expression of MLL2 was up-regulated in ESCC patients, and high expression of MLL2 was significantly correlated with worse clinical outcomes. MLL2 may play an oncogenic role as a negative prognostic factor for patients with ESCC. We also found that knockout of MLL2 not only inhibited the proliferation and migration, but also suppressed the EMT process of ESCC cells. These findings suggest that MLL2 may be used as a novel prognostic factor and therapeutic target for ESCC.
